# Corona-Krise: Was zeigt sich in der Herstellung und Bewältigung eines Ausnahmezustandes über die Möglichkeiten und Formen gesellschaftlicher Problembearbeitung?

**DOI:** 10.1007/s12054-020-00285-4

**Published:** 2020-04-09

**Authors:** Albert Scherr

**Affiliations:** Freiburg, Deutschland

**Keywords:** Corona, Ausnahmezustand, Krise, Moderne, Nationalismus

## Abstract

Der Beitrag setzt sich mit der Frage auseinander, was den gesellschaftlichen Reaktionen auf die Ausbreitung des Coronavirus zu Grunde liegt. In einer sozialwissenschaftlichen Perspektive wird argumentiert, dass diese nicht zureichend als zwangsläufige politische Konsequenz aus Einsichten der medizinischen Virenforschung verstanden werden können, sondern auch als Folge einer fundamentalen kulturellen Verunsicherung in den Blick zu nehmen sind. Aufgezeigt wird zudem die nationalistische Rahmung der politischen Reaktionen. Abschließend wird darauf auf die Gefahr hingewiesen, dass das, was in einem deklarierten temporären Ausnahmezustand gesellschaftlich durchsetzbar ist, auch unter anderen Bedingungen aktualisiert werden könnte.

Die Ausbreitung des Coronavirus wird als ein Ereignis wahrgenommen, das dazu zwingt, einen temporären Ausnahmezustand herbeizuführen. Zur Eindämmung der Krise sind einschneidende Maßnahmen wie Grenzschließungen und Kontaktverbote politisch durchgesetzt worden, die als unabweisbares Gebot der Vernunft erscheinen und deshalb auf große Akzeptanz stoßen. Dass sich eine Deutung der Situation als Ausnahmezustand ebenso etabliert hat wie die Überzeugung, dass dieser nur von begrenzter Dauer sein wird, ist jedoch keineswegs selbstverständlich, sondern voraussetzungsvoll und deshalb klärungsbedürftig. Klärungsbedürftig ist auch, mit welchen Entwicklungen nach einem Ende der Corona-Krise zu rechnen sein wird.^1^

Ausgangspunkt der folgenden Überlegungen ist eine Irritation: Wie ist zu erklären, dass die Ausbreitung des Coronavirus zu den bekannten drastischen politischen Maßnahmen geführt hat? Wie ist dies zu erklären, obwohl im März, als nationale Grenzen geschlossen, bundesweit der Betrieb an Kitas, Schulen und Hochschulen eingestellt und dann Kontaktverbote beschlossen wurden, deutlich weniger als 20.000 Menschen als Infizierte registriert waren und bei weniger als 50 Verstorbenen ein Zusammenhang zwischen ihrem Tod und einer Corona-Infektion festzustellen war? Auch wenn Vergleiche zu anderen Infektionskrankheiten aufgrund der nach wie vor hohen Unsicherheit über Infektionswege und Mortalitätsraten zweifellos problematisch sind, weisen sie dennoch darauf hin, dass es sich keineswegs um eine Reaktion handelt, die eingespielten, schon bislang gängigen Mustern folgt. Denn dass z. B. die Grippewelle 2017/18 ca. 25.000 Menschen in Deutschland das Leben kostete, hat nicht zu vergleichbaren Maßnahmen, noch nicht einmal zur Einführung einer Impfpflicht geführt.

Warum löst die Corona-Krise dagegen weltweit die Deklaration eines Ausnahmezustandes aus, der enorme staatliche Interventionsbereitschaften freisetzt und diesen gesellschaftliche Akzeptanz verschafft, während in anderen Fällen wesentlich gelassener auf bekannte Todesursachen – genauer: Faktoren, welche die Lebensdauer verkürzen – reagiert wird? Zur Verdeutlichung: Die WHO verzeichnet für 2018 1,4 bis 1,6 Mio. Tote in Folge von Tuberkulose, dies allerdings weit überwiegend außerhalb der entwickelten Gesellschaften des globalen Nordens (WHO 2019, S. 1). Aber auch die über 17.000 Todesfälle, die die Statistik für Deutschland (2017) als Folge infektiöser und parasitärer Krankheiten verzeichnet^2^, finden gewöhnlich keine öffentliche Aufmerksamkeit. Oder: Dass in Deutschland anhaltend jährlich 9000 bis 10.000 Todesfälle durch Suizide zu verzeichnen sind, führt weder zu massiver medialer Aufmerksamkeit, noch zu dringlichen Forderungen nach politischen Maßnahmen. Was also führt im Fall von Corona dazu, dass nunmehr von der Bundeskanzlerin eine den Zerstörungen des Zweiten Weltkriegs vergleichbare Herausforderung behauptet und mit pathetischer Übertreibung deklariert wird: „Wir sind eine Gemeinschaft, in der jedes Leben und jeder Mensch zählt“^3^?

Das verfügbare gesicherte Wissen um die Gefährlichkeit des Virus erklärt dies nicht, jedenfalls nicht zureichend. Denn die Letalität des Virus wird mit einiger Sicherheit auf maximal drei Prozent aller Infektionsfälle geschätzt, wobei zu berücksichtigen ist, dass tödliche Verläufe fast ausschließlich nur dann vorkommen, wenn altersbedingte Schwächen oder gravierende Vorerkrankungen hinzukommen. Unterscheidet dies Corona qualitativ z. B. von dem Infektionsrisiko, dem man sich ohnehin aussetzt, wenn man gezwungen ist, ein Krankenhaus aufzusuchen? Das Robert Koch-Institut geht diesbezüglich davon aus, dass für Deutschland von 400.000 bis 600.000 solcher Infektionen pro Jahr anzunehmen sind, die in 10.000 bis 20.000 Fällen zu Todesfällen führen (RKI 2019, S. 1). Die statistische Wahrscheinlichkeit eines tödlichen Verlaufs ist hier also ähnlich hoch.

## Überlastung und Kontrollverlust

Ein erster Ansatz, um die politischen Reaktionen auf den Virus zu verstehen, kann sich an dem orientieren, was an expliziten Gründen dafür genannt wird. Hingewiesen wurde diesbezüglich wiederkehrend auf eine drohende Überlastung des Gesundheitssystems, so etwa durch die Kanzlerin in ihrer Ansprache vom 18.03.2010: „Aber auch unsere Krankenhäuser wären völlig überfordert, wenn in kürzester Zeit zu viele Patienten eingeliefert würden, die einen schweren Verlauf der Corona-Infektion erleiden.“^4^

Dass hier von „unseren“ Krankenhäusern oder an anderer Stelle von „unserem Land“ und „unserer Gemeinschaft“ die Rede ist, verweist zunächst auf die Rahmung des Diskurses durch das, was Pogge (2011, S. 155 ff.) als nationenzentrierte Auffassung der Welt sowie als „gewöhnlichen“ bzw. „gehobenen Nationalismus“ kennzeichnet: Nationalstaaten sind in dieser Perspektive nicht allein politische Organisationsformen, sondern moralische Gemeinschaften, die eine über Familien, Verwandtschaften oder Religionen hinausreichende Empathie und Verantwortlichkeit innerhalb der Nationalgesellschaft etablieren, diese aber zugleich nach außen hin, gegenüber Bürger_innen anderer Nationalgesellschaften, begrenzen. Dem entspricht ein Primat der politischen Verantwortlichkeit für die Problemlagen und Interessen der Bürger_innen der eigenen Nationalgesellschaft. Die Reaktionen auf Corona, so die sich als selbstverständlich inszenierende Etablierung einer Zuständigkeit nationaler Regierungen für ihre Bürger_innen, wo immer diese sich weltweit auch gerade befinden (Rückholung), die Schließung nationaler Grenzen (statt z. B. regionaler Abschottungen) zeigen insofern an, dass die Zeiten der Nationalstaates als Denkhorizont, als primäre politische Handlungseinheit und als weitgehend exklusive moralische Gemeinschaft längst nicht überwunden sind.

Corona ist so betrachtet auch eine Krise der Globalisierung – im Sinne einer Krise der fortschreitenden Etablierung supranationaler moralischer Verantwortlichkeit und politischer Zuständigkeit. Immerhin aber werden punktuell auch nationale Grenzen überschreitende Handlungsbereitschaften sichtbar, so bei internationalen Hilfslieferungen medizinischer Güter und bei der Aufnahme französischer und italienischer Patient_innen in deutsche Krankenhäuser, nicht aber (oder jedenfalls nur sehr begrenzt) im Umgang mit Flüchtlingen an den europäischen Außengrenzen oder auf den griechischen Inseln.

Entscheidender ist im vorliegenden Zusammenhang aber, dass in einschlägigen politischen Erklärungen nicht näher erläutert wird, worin die unbedingt zu vermeidende Überlastung des Gesundheitssystems besteht. Dies wurde bislang nicht nur durch die Kanzlerin nicht näher erläutert. Diesbezüglich liegt es m. E. nahe zu vermuten, dass die Vorstellung, dass Menschen mit lebensbedrohlichen Symptomen in Deutschland (und anderen hoch entwickelten Ländern des globalen Nordens) nicht auf dem Stand der modernen medizinischen Technik behandelt werden können – was bekanntlich in weiten Teil der Welt Normalität ist – normativ als inakzeptabel gilt sowie mit Befürchtungen dahingehend einhergeht, dass dies nicht nur zu einem Reputationsverlust der Politik, sondern auch zu irrationalen Reaktionen und sozialen Konflikten in Teilen der Bevölkerung führen könnte. Denn eine Politik, die öffentlich eingestehen müsste, die Bürger_innen nicht mehr mit den Mitteln moderner Medizin vor einem Virus schützen oder daran Erkrankte behandeln zu können, würde damit eingestehen, dem für moderne Staaten grundlegenden Sicherheitsversprechen nicht entsprechen zu können. Und die Vorstellung, was geschehen könnte, wenn schwer Erkrankte durch deutsche Krankenhäuser abgewiesen werden müssten – Bürger_innen also nicht mehr auf staatliche Gewährleistung von Hilfen vertrauen könnten – setzt dystopische Fantasien frei.

Folgt man dieser Einschätzung, dann besteht die befürchtete Überlastung des Gesundheitssystems nicht allein darin, dass dieses an Kapazitätsgrenzen geraten könnte, sondern auch in einem möglichen politischen Reputationsverlust sowie einem Kontrollverlust bezüglich der sozialen Folgen. Eine unausgesprochene – und zur Vermeidung einer selbstverstärkenden Dynamik auch unaussprechbare – Befürchtung könnte darin bestehen, dass chaotische soziale Zustände zu befürchten sind, die weder durch ordnungspolitische Maßnahmen, noch durch Appelle an Solidarität und Selbstdisziplinierung beherrschbar wären. Denn selbst bei kleineren Krisensituationen – etwa punktuellen Zusammenbrüchen des Bahnverkehrs – ist immer wieder erfahrbar, dass ein gemeinsamer solidarischer Umgang mit der Situation immer in der Gefahr steht, in egoistische und aggressive Formen der individuellen Selbstbehauptung umzukippen. So betrachtet ist es politisch klug, an Solidarität zu appellieren und zu vermeiden versuchen, dass ein Vertrauensverlust in die Leistungsfähigkeit des Gesundheitssystems und die Steuerungskapazitäten des politischen Systems entsteht. All dies aber geschieht auf einer bislang unsicheren Basis, denn das Ausmaß und die Dauer der Bedrohung sind nur begrenzt einschätzbar.

## Verunsicherung der modernen Illusion

Kultursoziologisch liegt ein darüber hinausgehender, grundsätzlicherer Erklärungsansatz nahe: Das grundlegende Versprechen der Moderne ist das der Errichtung einer geordneten Gesellschaft, die auf der Grundlage rationalen Wissens sichere Lebensverhältnisse ermöglicht. Von der wissenschaftlichen Vernunft und der staatlichen Ordnung wird erwartet, Gefahren in begrenzte und kontrollierbare Risiken verwandeln zu können, diffuse Ängste vor drohendem Unheil durch Wahrscheinlichkeitskalküle zu rationalisieren (Luhmann 1991). Der moderne Staat etabliert sich dem entsprechend als sorgender Staat, in dessen Zuständigkeit neben der Armenfürsorge und der Kriminalitätskontrolle auch Maßnahmen der Hygiene fallen, durch die eine Ausbreitung ansteckender Krankheiten vermieden werden soll (de Swaan 1993). Undurchschaubare und mit Mitteln der wissenschaftlich-technischen Vernunft nicht fassbare und nicht beherrschbare Gefahren sind im Selbstverständnis der modernen Gesellschaft nicht vorgesehen. Kennzeichen der Moderne ist Max Weber (1917/1999, S. 594) zu Folge vielmehr der Glaube daran, dass es „prinzipiell keine geheimnisvollen unberechenbaren Mächte gebe (…), daß man vielmehr alle Dinge – im Prinzip – durch Berechnen beherrschen könne“. Bereits Max Weber – und daran anschließend u. a. Horkheimer und Adorno in der Dialektik der Aufklärung (1944/1968) sowie Zygmunt Bauman (1992) – äußern jedoch fundamentale Zweifel an der Tragfähigkeit dieser zweifellos wirkungsmächtigen Illusion und fordern zur Analyse der problematischen Folgen des Versuchs einer umfassenden rationalen Natur- und Menschenbeherrschung auf.

Dem Rationalitätsglauben der Moderne entspricht, dass tödliche, mit den zeitgenössisch verfügbaren Mitteln unerklärbare und nicht beherrschbare Seuchen, wie die mittelalterliche Pest und die frühmodernen Cholera-Epidemien, im bislang vorherrschenden Selbstverständnis der Moderne als historische Phänomene betrachtet werden, die durch den medizinischen und technischen Fortschritt überwunden sind. Dies ist der Fall, obwohl auch noch Anfang des 20. Jahrhunderts mit der sog. spanischen Grippe, an der 1918 und 1919 ca. 50 Mio. Menschen gestorben sind, ein vergleichbares Phänomen aufgetreten ist. Dies ist jedoch aus dem gesellschaftlichen Gedächtnis verdrängt worden (Honigsbaum 2020).

Handelte es sich bei der spanischen Grippe um ein ungleichzeitiges Phänomen, um ein letztes Auftreten eines vormodernen Phänomens in der Moderne, ein nicht mehr bedeutsames letztes Aufleuchten vergangener Zeiten? McNeill (1976) kommt in seiner wichtigen Studie zur Bedeutung von Epidemien für die Menschheitsgeschichte zu einer anderen Einschätzung. Grundlage dafür ist die Annahme, dass die Koevolution von Menschen, Tieren und Mikroparasiten immer wieder dazu führt, dass sich Viren entwickeln, denen der menschliche Organismus noch nicht angepasst ist. Seine Folgerung lautet: „Einfallsreichtum, Wissen und Organisation verändern dies, aber sie können die Verwundbarkeit der Menschheit gegenüber der Invasion durch parasitäre Formen des Lebens nicht aufheben. Infektionskrankheiten, die die Entstehung der Menschheit begleitet haben, werden so lange dauern wie die Menschheit selbst und werden sicherlich, wie bisher, einer der grundlegenden Parameter und Bestimmungsfaktoren der menschlichen Geschichte bleiben.“ (McNeill 1976, S. 257)

Die Ausbreitung von Ebola und Aids sind prominente Fälle, die als Bestätigung dieser Einschätzung, als Beleg für eine auch mit modernen Mitteln nicht aufhebbare Vulnerablität, gelten können. Im Unterschied dazu kann für Corona jedoch erstens festgestellt werden, dass – wie etwa bei Ebola – keine lokale Eingrenzung auf die Regionen des subsaharischen Afrika gegeben ist, die ohnehin nicht als Bestandteil der westlichen Moderne wahrgenommen werden, und für die das Vorhandensein tödlicher Tropenkrankheiten und deren unzureichende medizinische Behandlung als akzeptabler Normalzustand gilt. Zweitens gilt, im Unterschied zu Aids, dass kein Zusammenhang mit individuell zurechenbarem undiszipliniertem Sexualverhalten besteht, sondern potenziell jede_r, auch normkonforme Bürger_innen der westlichen Wohlstandsgesellschaften, gefährdet ist.

Durch Corona ist die moderne Gesellschaft damit in einen vormodernen Zustand zurückversetzt, in den Zustand einer unbeherrschbaren Bedrohung, während zugleich vormoderne Deutungen dafür – als göttliche Strafe, als hinzunehmende Übermacht der Naturgewalten, als Folge des Wirkens böser Mächte – und diesen entsprechende Bewältigungsformen obsolet sind. Gleichzeitig aber gelingt es modernen Gesellschaften, sich – jedenfalls gegenwärtig noch – damit zu beruhigen, dass es sich um einen bloß temporären Ausnahmezustand handelt, um ein Problem, dessen gesundheitliche, ökonomische und soziale Auswirkungen mit modernen Mitteln beherrschbar bleiben.^5^ Es ist zugleich offenkundig, dass die nunmehr etablierten Maßnahmen der Eingrenzung und Kontrolle nur auf begrenzte Zeit durchhaltbar sind, die ergriffenen Maßnahmen also von der Hoffnung getragen sind, dass es in absehbarer Zeit möglich sein wird, Corona mit dem Mittel der technisch-wissenschaftlichen Naturbeherrschung präventiv oder therapeutisch zu beherrschen. Nur deshalb ist eine Deutung als Ausnahmezustand – und nicht als Zusammenbruch des Gesellschafts- und Kulturmodells der westlichen Moderne – plausibel.

Sollte dies jedoch nicht der Fall sein – was im modernen Denken ausgeschlossen, aber nicht prinzipiell ausschließbar ist – wäre nicht nur mit steigenden Todeszahlen, sondern auch mit katastrophalen wirtschaftlichen, sozialen und politischen Entwicklungen zu rechnen, nicht zuletzt auch einem Erstarken irrationaler Deutungs- und Bewältigungsformen. Darin, dass die Waffenverkäufe seit Ausbreitung des Virus in den USA steigen, nicht zuletzt durch Käufe von Amerikanern mit asiatischem Aussehen, die mit der Gefahr von rassistischen Schuldzuweisungen und Übergriffen rechnen, deutet sich exemplarisch an, wozu eine länger anhaltende Krise führen könnte. Und auch in Deutschland ist schwer absehbar, womit zu rechnen wäre, wenn sich die wirtschaftliche Krise verschärfen und Versorgungsengpässe bei Grundbedarfen auftreten würden, deren Ende nicht absehbar ist. Es gibt auch deshalb gute Gründe, die Hoffnung auf eine zeitliche Begrenzbarkeit des Ausnahmezustandes nicht aufzugeben.

## Nach Corona: Konflikte um Deutungs- und Handlungsmacht

Davon ausgehend, dass keine Katastrophe eintreten wird, sondern sozialhygienische Maßnahmen zu einem Abflachen der Infektionsrate führen sowie Impfstoffe und Therapien zur Verfügung stehen werden, sind gleichwohl erhebliche gesellschaftliche Konsequenzen zu erwarten. Durchaus zweifelhaft ist aber, ob nach Corona nichts mehr so sein wird wie zuvor. Denn erstens scheinen sich eingespielte gesellschaftliche Reaktionsmuster – so die Zuweisung der zentralen Verantwortlichkeit an nationalstaatliche Politik, die Einschränkung bürgerlicher Freiheiten im Fall äußerer Bedrohungen, das medizinische Paradigma der Ursachenforschung und Intervention, die Mobilisierung privatwirtschaftlicher Ressourcen für die Entwicklung innovativer Medikamente – zu bewähren, oder erweisen sich als alternativlos. Zweitens wäre es soziologisch betrachtet sehr naiv, von eindeutigen und unabweisbaren Lehren aus der Krise auszugehen, die zwingend zu bestimmten gesellschaftlichen Konsequenzen führen.

Erwartbar sind vielmehr Konflikte um konkurrierende Deutungen und ihre politischen Implikationen. Globalisierungskritiker werden die Krise als Beweis für die Unverzichtbarkeit nationaler Grenzen darstellen und darin einen weiteren Grund für ihre Kritik wirtschaftlicher, politischer und kultureller Globalisierung sehen. Kapitalismuskritisch wird dagegen bereits jetzt eine erwartbare Schuldzuweisung formuliert und eine transnationale Einhegung der Ökonomie eingefordert.^6^ Anhänger eines starken Sicherheitsstaates werden sich in der Überzeugung bestätigt sehen, dass es prinzipiell erforderlich sein kann, Freiheitsrechte in Notlagen einzuschränken. Kritiker_innen des Neoliberalismus werden für eine Stärkung des Sozialstaates, insbesondere den Ausbau der staatlichen Finanzierung des Gesundheitssystems, plädieren. Liberale Ökonomien werden auf die Leistungsfähigkeit marktwirtschaftlicher Innovationen verweisen, sobald eine der einschlägigen Firmen ein geeignetes Medikament entwickelt hat. Esoteriker_innen und Verschwörungstheoretiker_innen aller Richtungen werden ihrer Kreativität freien Lauf lassen, usw.

Der Ausgang dieser Deutungskämpfe und politischen Konflikte ist nicht absehbar. Zu befürchten ist allerdings, dass sich der Einfluss nationalistischer und autoritärer Gesellschaftsmodelle verstärken wird. Denn in den bisherigen Krisenreaktionen war keine wirksame Gegentendenz zur Dominanz nationaler Zuständigkeiten und Verantwortlichkeiten zu vernehmen, Solidarität wurde primär als Solidarität innerhalb von Nationalgesellschaften eingefordert und realisiert. Und Kritiker_innen von staatlichen Freiheitseinschränkungen werden erhebliche Mühe haben, in der wiederhergestellten Normalität gesellschaftlichen Rückhalt für die Forderung zu finden, den Staat wieder in jene Grenzen zurückzuverweisen, die ihm vor dem Ausnahmezustand auferlegt waren. Denn das im Fall der Epidemie – mit guten Gründen – akzeptierte Konzept der Freiheitseinschränkungen könnte künftig auch in anderen Situationen wiederbelebt werden, die aus anderen Gründen politisch als bedrohliche Krisen und Ausnahmezustände definiert werden.

## Footnotes

1 Dieser Beitrag wurde im März 2020 verfasst. Seitdem eingetretene Entwicklungen konnten also nicht mehr berücksichtigt werden.

2 https://www.destatis.de/DE/Themen/Gesellschaft-Umwelt/Gesundheit/Todesursachen/Tabellen/gestorbene_anzahl.html.

3 https://www.bundeskanzlerin.de/bkin-de/aktuelles/fernsehansprache-von-bundeskanzlerin-angela-merkel-1732134.

4 https://www.bundeskanzlerin.de/bkin-de/aktuelles/fernsehansprache-von-bundeskanzlerin-angela-merkel-1732134.

5 Staatliche Maßnahmen der sozialen Kontrolle und Sozialhygiene waren bereits in der frühen Moderne erfolgreiche Mittel gegen die Ausbreitung von Infektionskrankheiten in den entstehenden Großstädten (de Swaan 1993, S. 134 ff..).

6 So etwa von Illouz in einem Interview mit der Süddeutschen Zeitung vom 24.03.2019: „Unsere Gesellschaften waren zu sehr damit beschäftigt, nach Gewinn zu streben, Land und Arbeit auszubeuten, wo immer sie konnten. In einer Welt nach Corona werden wir zoonotische Infektionen und chinesische Schlachtermärkte zu einem Problem der Völkergemeinschaft machen müssen. Wenn sich das iranische Nukleararsenal überwachen lässt, gibt es keinen Grund, warum wir nicht eine internationale Überwachung der Quellen zoonotischer Infektionen fordern sollten. Die Unternehmerschaft auf der ganzen Welt wird vielleicht endlich begreifen, dass es erst einmal eine Welt geben muss, bevor man sie ausbeuten kann.“ Was allerdings chinesische Wildtiermärkte mit Kapitalismus zu tun haben, bleibt dabei ebenso unklar wie die Bedingungen der Bereitschaft Chinas, eine internationale Überwachung zu akzeptieren.
